# AFP ratio predicts HCC recurrence after liver transplantation

**DOI:** 10.1371/journal.pone.0235576

**Published:** 2020-07-02

**Authors:** Christine Koch, Theresa Bette, Oliver Waidmann, Natalie Filmann, Christopher Schrecker, Jörg Trojan, Nina Weiler, Johannes Vermehren, Andreas A. Schnitzbauer, Wolf Otto Bechstein, Stefan Zeuzem, Eva Herrmann, Martin-Walter Welker

**Affiliations:** 1 Medizinische Klinik 1, Universitätsklinikum Frankfurt, Frankfurt, Germany; 2 Institut für Biostatistik und Mathematische Modellierung, Universitätsklinikum Frankfurt, Frankfurt, Germany; 3 Klinik für Allgemein- und Viszeralchirurgie, Universitätsklinikum Frankfurt, Frankfurt, Germany; Texas A&M University, UNITED STATES

## Abstract

**Background/aims:**

Hepatocellular carcinoma (HCC) is a leading indication for liver transplantation (LT) worldwide. Early identification of patients at risk for HCC recurrence is of paramount importance since early treatment of recurrent HCC after LT may be associated with increased survival. We evaluated incidence of and predictors for HCC recurrence, with a focus on the course of AFP levels.

**Methods:**

We performed a retrospective, single-center study of 99 HCC patients who underwent LT between January 28^th^, 1997 and May 11^th^, 2016. A multi-stage proportional hazards model with three stages was used to evaluate potential predictive markers, both by univariate and multivariable analysis, for influences on 1) recurrence after transplantation, 2) mortality without HCC recurrence, and 3) mortality after recurrence.

**Results:**

19/99 HCC patients showed recurrence after LT. Waiting time was not associated with overall HCC recurrence (HR = 1, p = 0.979). Similarly, waiting time did not affect mortality in LT recipients both with (HR = 0.97, p = 0.282) or without (HR = 0.99, p = 0.685) HCC recurrence. Log_10_-transformed AFP values at the time of LT (HR 1.75, p = 0.023) as well as after LT (HR 2.07, p = 0.037) were significantly associated with recurrence. Median survival in patients with a ratio (AFP at recurrence divided by AFP 3 months before recurrence) of 0.5 was greater than 70 months, as compared to a median of only 8 months in patients with a ratio of 5.

**Conclusion:**

A rise in AFP levels rather than an absolute threshold could help to identify patients at short-term risk for HCC recurrence post LT, which may allow intensification of the surveillance strategy on an individualized basis.

## Introduction

Hepatocellular carcinoma (HCC) is a leading indication for liver transplantation (LT) in East Asia, Europe, and North America [1–3]. The overall five-year survival rate of patients with HCC is comparable to non-malignancy indications, when tumor size is limited at the time of LT [4,5]. The Milan criteria are commonly used to decide whether patients with HCC are eligible for LT or not, and are defined as a single HCC nodule not exceeding 5 cm, or a maximum of three nodules not exceeding 3 cm each [5]. While some studies have investigated whether transplantation beyond the Milan criteria is feasible [6–8], HCC recurrence after LT is still a major concern even when the Milan criteria are fulfilled [9]. Despite general efforts and advances in the treatment of HCC in recent years [10], treatment of recurrent HCC after LT remains a challenge due to the lack of prospective, controlled studies addressing this issue [11]. Hence, early identification of patients at risk for HCC recurrence is of paramount importance.

Currently, there is no clear guideline for follow-up and surveillance of patients after LT for HCC [12]. It would be desirable to predict the individual risk for HCC recurrence more accurately, thereby reducing the need for repeated radiation exposure and the use of contrast agents. In recent years, a number of donor-related and transplant-related risk factors for tumor development besides immunosuppression have been described, to facilitate identification of patients at risk for an early recurrence [13–15]. Alpha-fetoprotein (AFP) is an established and routine tumor marker in patients with HCC, which is readily available for patients who were AFP-positive before LT. Of note, AFP values >1000 ng/ml before LT have been associated with the risk of HCC recurrence after LT [16–18]. High AFP serum levels may be a surrogate parameter for vascular infiltration, a well-characterized predictor for HCC recurrence after LT [19]. However, the clinical value of AFP in HCC surveillance after LT has not been closely investigated.

In the current study, we evaluated the incidence of and predictors for HCC recurrence (with a focus on the course of AFP levels) in liver graft recipients, who suffered from HCC prior to LT and were transplanted within the Milan criteria, in a high MELD region with correspondingly longer waiting times.

## Methods

### Study design

The aim of this retrospective, single-center study was to investigate the recurrence rate of HCC after LT at a German liver transplant center and to analyze predictors for HCC recurrence. Patients transplanted elsewhere were included if they participated in our clinic’s liver post-transplant surveillance program and if sufficient data were available. The study was approved by the institutional review board (internal reference number 268/13-006) of the University Hospital Frankfurt. Informed consent to participate in the local liver transplant registry was obtained from all patients alive at the time of the study. In accordance with legal requirements and ethics committee vote, data of deceased patients were also included in the analysis. Inclusion criteria of the registry were a history of liver transplantation for any indication and age older than 18 years. Patients with HCC before liver transplantation were identified from the registry and the respective data sets were transferred to a separate database, which was the basis for further analyses as reported here. Finally, all data were pseudonymized for analysis and only non-identifiable data were published. Data from individual patients may have been reported previously with respect to different topics [20–22].

### Patient data

The study database was based on local electronic health records including epidemiological data, age, body mass index (BMI), overall and recurrence-free survival with regard to HCC after LT and, if applicable, dates of death. Diagnosis of HCC recurrence after LT was confirmed by radiographic and/or histopathologic examination. Laboratory data analyzed included AFP serum values, as well as virological parameters including hepatitis B/C and CMV status. Finally, a medication history including details of immunosuppression and a full medical history were obtained. Data closure and end of follow-up was February 11^th^, 2019.

### Statistical analyses

Clinical and biochemical patient characteristics were expressed as mean ± standard deviation (SD) or median and range, as appropriate. A multi-stage proportional hazards model with three stages was used to evaluate potential predictive markers, both by univariate and multivariable analysis, for influences on 1) recurrence after transplantation, 2) mortality without HCC recurrence, and 3) mortality after recurrence. Endpoints of the regression analysis were recurrence and death, and these were analyzed as strata in the multi-stage model. HCC recurrence was analyzed as a factor with proportional influence on death and was also included in the otherwise univariate analysis of the multi-stage model. When analyzing AFP as a predictor for recurrence and mortality, only values transformed to log_10_(1+AFP) were included in the regression model. Furthermore, the normal range of AFP kinetics in patients without recurrence was described by empirical 90% quantiles. Software: R with the packages “survival” and “mstate” (R Foundation for Statistical Computing, Vienna, Austria).

## Results

### Patient characteristics and incidence of HCC recurrence

A total of 99 patients who underwent LT for HCC between January 28^th^, 1997 and May 11^th^, 2016 were included in the current study. Of these 99 patients, 22 were transplanted between 1997 and 2006. 19/99 patients suffered an HCC recurrence in the observation period. Detailed patient characteristics are shown in Table 1. The mean waiting time between HCC diagnosis and transplantation was 12.0 ± 9.3 months (median 12.0 months, IQR 5.5–15.5). Waiting time was not associated with overall HCC recurrence (HR = 1, p = 0.979). Similarly, waiting time did not affect mortality in LT recipients both with (HR = 0.97, p = 0.282) or without (HR = 0.99, p = 0.685) HCC recurrence.

**Table 1:** Patient baseline characteristics (at LT).

	all patients (n = 99)	influence on recurrence		influence on mortality without recurrence		influence on mortality after recurrence	
		HR1	p-value	HR2	p-value	HR3	p-value
**female gender, n (%)**	31/99 (31.3%)	0.53	0.256	1.20	0.652	0.66	0.537
**age [y], mean (SD)**	55.6 (6.7)	1.01	0.774	1.03	0.283	1.05	0.416
**median (IQR)**	56.0 (52.0–60.0)						
**BMI [kg/m**^**2**^**], mean (SD)**	26.6 (5.2)	1.03	0.503	0.99	0.786	1.08	0.156
**median (IQR)**	26.4 (23.1–29.1)						
**etiology of liver disease, n (%)**							
**viral hepatitis only**	65/99 (65.7%)	Ref		Ref		Ref	
**Alcohol**	17/99 (17.2%)	0.56	0.442	0.74	0.591	3.13	0.178
**viral hepatitis and alcohol**	7/99 (7.1%)	2.35	0.183	n.a.	n.a.	3.95	0.074
**alcohol and other**	2/99 (2.0%)	n.a.	n.a.	3.44	.099	n.a.	n.a.
**Other**	8/99 (8.1%)	0.55	0.565	0.40	0.369	3.44	0.285
**waiting time (months), mean (SD)**	12.0 (9.3)	1.00	0.979	0.99	0.658	0.97	0.282
**median (IQR)**	12.0 (5.5–15.5)						
**labMeld before LT, mean (SD)**	13.5 (6.2)	0.96	0.392	0.99	0.863	1.07	0.094
**median (IQR)**	12.0 (8.8–15.0)						
**SE-Meld before LT, mean (SD)**	28.9 (3.4)	0.87	0.076	0.96	0.575	1.16	0.263
**median (IQR)**	29.0 (28.0–31.0)						
**SE criteria at listing, n (%)**	52/84 (61.9%)	0.59	0.271	1.32	0.574	0.38	0.060
**SE criteria at LT, n (%)**	69/86 (80.2%)	0.62	0.366	0.58	0.301	0.59	0.328
**Milan criteria at initial diagnosis, n (%)**	63/77 (81.8%)	0.33	**0.033**	1.49	0.596	0.44	0.141
**Milan criteria at LT, n (%)**	75/85 (88.2%)	0.33	0.058	0.65	0.490	0.60	0.397
**HBsAg positive, n (%)**	7/82 (8.5%)	0.87	0.897	1.09	0.911	n.a.	n.a.
**anti-HBc positive, n (%)**	44/82 (53.7%)	1.88	0.248	1.74	0.275	1.485	0.562
**anti-HIV positive, n (%)**	2/58 (3.4%)	9.56	**0.045**	n.a.	n.a.	n.a.	n.a.
**anti-CMV positive, n (%)**	68/89 (76.4%)	4.22	0.163	0.60	0.261	0.88	0.907
**diabetes mellitus, n (%)**	34/98 (34.7%)	1.41	0.462	0.90	0.810	0.82	0.687
**HbA1c [%], mean (SD)**	5.7 (1.2)	1.25	0.449	1.00	0.992	1.48	0.493
**median (IQR)**	5.4 (4.9–6.4)						
**CKD, n (%)**	48/99 (48.5%)	0.68	0.415	1.12	0.787	0.72	0.504
AFP [ng/mL], mean (SD)*	1166.7 (7448.6)	2.25	**<0.001**	0.89	0.749	0.85	0.419
**median (IQR)**	8.2 (4.5–25.7)						
**GFR MDRD [ml/min], mean(sd)**	84.8 (26.1)	1.00	0.735	1.00	0.692	0.98	0.255
**median (IQR)**	83.2 (71.2–102.3)						
**dialysis, n (%)**	2/90 (2.2%)	2.16	0.449	n.a.	n.a.	n.a.	n.a.
**grade, n (%)**							
**G1**	19/78 (24.4%)	Ref		Ref		Ref	
**G1-2**	7/78 (9.0%)	n.a.	n.a.	0.63	0.680	n.a.	n.a.
**G2**	47/78 (60.3%)	2.93	0.158	1.39	0.573	0.91	0.902
**G3**	5/78 (6.4%)	49.51	**<0.001**	6.71	0.111	0.61	0.590
**T stage, n (%)**							
**T0**	25/96 (26.0%)	Ref		Ref		Ref	
**T1**	31/96 (32.3%)	n.a.	n.a.	1.04	0.937	n.a.	n.a.
**T2**	27/96 (28.1%)	8.53	**0.043**	1.71	0.309	n.a.	n.a.
**T3**	13/96 (13.5%)	30.50	**0.001**	0.57	0.603	n.a.	n.a.
**Milan criteria in explanted liver, n (%)**	65/94 (69.1%)	0.09	**<0.001**	0.77	0.570	0.24	0.060
**vascular infiltration, n (%)**	11/93 (11.8%)	11.39	**<0.001**	1.60	0.532	1.77	0.255
**necrotic tumor, n (%)**	12/95 (12.6%)	0.38	0.352	1.06	0.920	n.a.	n.a.

* Log10 transformation for survival analysis

### Clinical predictors associated with HCC recurrence and mortality with and without HCC recurrence

A thorough overview of the risk analysis for HCC recurrence is given in Table 1. In detail, larger tumor size (T2 vs. T0, HR = 8.53, p = 0.043; T3 vs. T0, HR 30.5, p = 0.001), vascular infiltration (HR = 11.39, p<0.001), and higher AFP values (log_10_-transformed; HR = 2.25 for a factor of 10, p<0.001) were significantly associated with HCC recurrence. A tumor stage within the Milan criteria markedly reduced the risk of recurrence (HR = 0.09, p<0.001). With regard to overall survival, HCC recurrence was strongly significant (p<0.0001), and was adjusted for in the analysis of the remaining baseline parameters shown in Tables 1 and 2. Here, only transplantation of a split liver graft (with limited case numbers, n = 5, HR = 6.56, p = 0.004) was associated with impaired survival after LT. Multivariate analysis (Table 3) confirmed tumor size (T2/T3), tumor grade (G3), and hospital-based allocation to be associated with risk for recurrence.

**Table 2:** Transplant and donor characteristics.

	all patients (n = 99)	influence on recurrence		influence on mortality without recurrence		influence on mortality after recurrence	
		HR1	p-value	HR2	p-value	HR3	p-value
**living donor, n (%)**	4/98 (4.1%)	1.74	0.593	4.07	0.062	0.98	0.982
**split liver graft, n (%)**	5/98 (5.1%)	1.67	0.618	6.56	**0.004**	0.98	0.987
**female donor, n (%)**	41/95 (43.2%)	1.18	0.722	0.59	0.221	0.70	0.484
**age donor [y], mean (SD)**	54.9 (18.7)	1.01	0.474	0.99	0.432	1.01	0.378
**median (IQR)**	56.0 (47.0–70.0)						
**anti-HBc donor, n (%)**	2/13 (15.4%)	n.a.	n.a.	2.65	0.496	n.a.	n.a.
**CMV positive donor, n (%)**	60/85 (70.6%)	0.85	0.745	1.95	0.290	1.79	0.290
**hospital-based allocation, n (%)**	3/98 (3.1%)	6.99	**0.012**	3.86	0.197	1.54	0.575
**CIT [h], mean (SD)**	9.4 (2.8)	0.94	0.483	0.93	0.271	1.07	0.483
**median (IQR)**	9.8 (8.1–11.2)						

**Table 3:** multivariate analysis of predictors for HCC recurrence after LT.

	Beta	SE	HR (95% CI)	p-value
**T stage T2**	2.50	1.07	12.2 (1.50–99.8)	0.0193
**T stage T3**	3.75	1.07	42.6 (5.15–352.4)	0.0005
**Grade G3**	2.33	1.73	10.3 (2.48–42.8)	0.0013
**hospital-based allocation**	-2.28	0.85	0.10 (0.02–0.54)	0.0074

### AFP for prediction of HCC recurrence

Based on the results of the univariate analysis, we next sought to further investigate the role of AFP as a possible predictive marker for HCC recurrence and mortality as competing endpoints.

**Fig 1:**
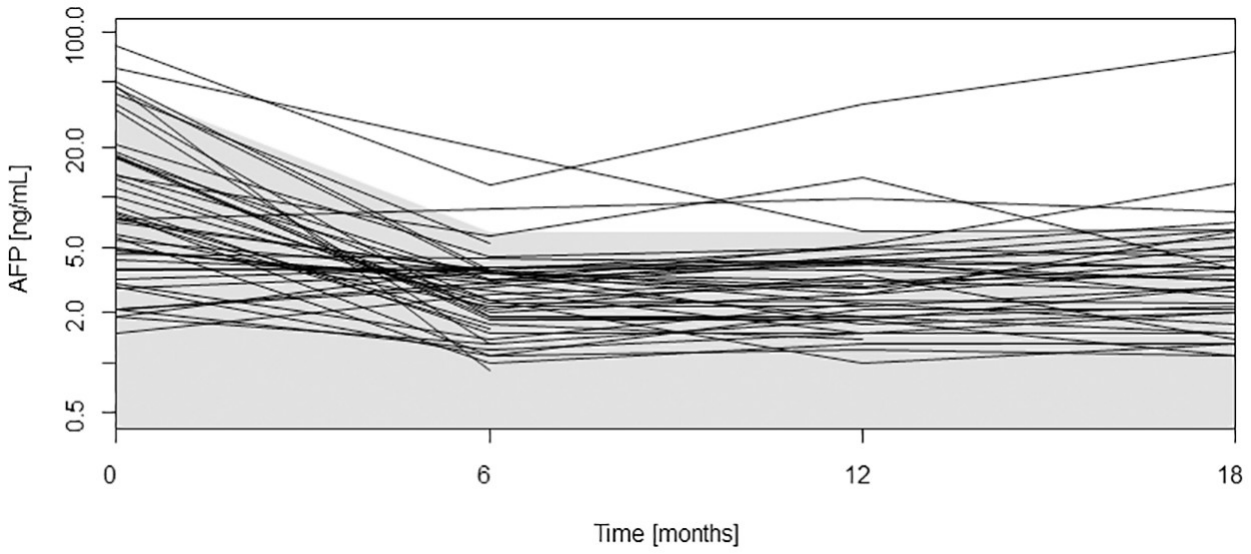
The AFP values within the first 18 months in patients, who survived at least 24 months and who did not develop recurrent HCC in this time period, are given. The shaded area shows the 90% percentile for AFP values (below 46.8 ng/ml at LT and below 6.27 ng/ml from months 6 through 18 after LT).

In 90% of patients who survived at least 24 months without HCC recurrence, AFP levels were below 46.8 ng/ml at LT, and below 6.27 ng/ml from months 6 through 18 (Fig 1).

**Fig 2:**
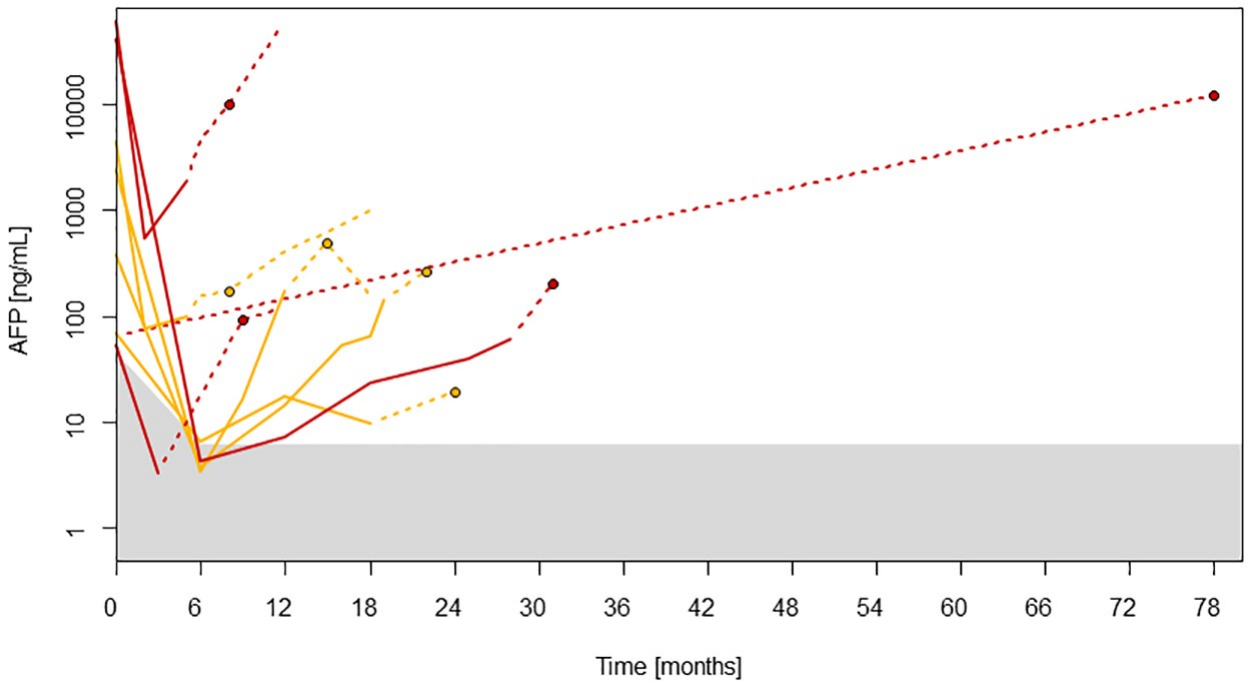
To illustrate AFP values of patients with HCC recurrence and AFP values above 50 ng/mL at LT, solid lines are used to indicate the course of AFP values until the last available measurement 3 months before diagnosis of recurrence, while the further course of AFP levels until diagnosis of HCC recurrence and thereafter is shown by dotted lines. Red lines and dots: patients with HCC recurrence in the liver; orange lines and dots: extrahepatic recurrence; dots: diagnosis of recurrence; shaded area: 90% percentile for non-recurrence.

We next divided patients who suffered from HCC recurrence after LT in two groups based on AFP levels at LT. The first group consisted of patients with AFP values above 50 ng/ml at LT. Of these, all patients except one had AFP levels above 6.27 ng/ml (90% percentile for non-recurrence from months 6 through 18 after LT) already 3 months before HCC recurrence (Fig 2).

**Fig 3:**
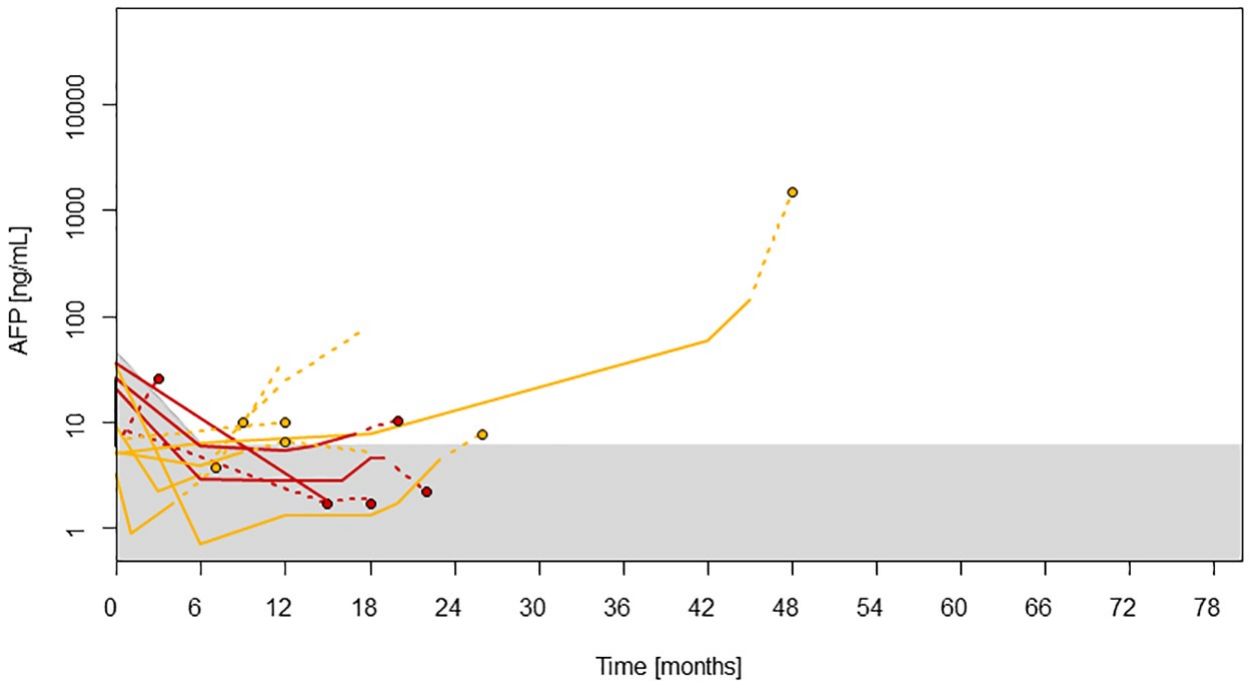
This graph shows the AFP values of patients with HCC recurrence and AFP values below 50 ng/mL at LT. To focus on information available for recurrence prediction before diagnosis, solid lines are again used to illustrate the course of AFP values until the last available measurement 3 months before diagnosis of recurrence. Further course of AFP until diagnosis of recurrence and thereafter is shown by dotted lines. Red lines and dots: patients with HCC recurrence in the liver; orange lines and dots: extrahepatic recurrence; dots: diagnosis of recurrence; shaded area: 90% percentile for non-recurrence.

In contrast, only two of the patients with HCC recurrence and AFP levels below 50 ng/ml at LT showed AFP values above the 90% percentile already 3 months before HCC recurrence (Fig 3).

Since we found these differences between patients with AFP values above or below 50 ng/ml at the time of LT, we developed a statistical multivariate multi-stage model with AFP as a time-dependent variable. AFP at the time of LT, and AFP after LT, were compared as factors influencing HCC recurrence. We found that log_10_-transformed AFP values were significantly associated with recurrence at the time of LT (HR 1.75, p = 0.023) as well as after LT (HR 2.07, p = 0.037).

### AFP for prediction of survival after HCC recurrence

**Fig 4:**
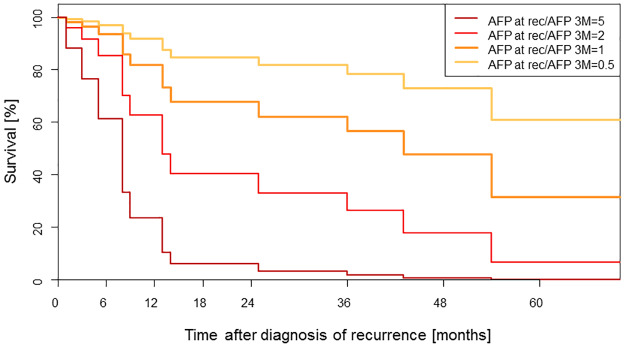
Prediction of survival after diagnosis of recurrence depending on AFP levels (p = 0.0232).

To determine the influence of different AFP values on survival after recurrence, we compared AFP levels at LT as well as 6 and 3 months before recurrence, at recurrence and after recurrence in a multivariate model. We found that the ratio of AFP at recurrence to AFP 3 months before recurrence was predictive for survival following HCC recurrence. Mortality risk was increased if AFP values were high at recurrence, and/or low 3 months before recurrence. Fig 4 shows the prediction of survival following HCC recurrence depending on the AFP ratio (p = 0.0232). Median survival in patients with a ratio (AFP at recurrence divided by AFP 3 months before recurrence) of 0.5 was greater than 70 months, as compared to a median of only 8 months in patients with a ratio of 5.

### Clinical course and treatment of patients with HCC recurrence

Treatment of recurrent HCC after LT was done on a case by case basis, considering the individual patient´s performance status, liver graft function, and disease extent (metastatic pattern). Management decisions were made in an interdisciplinary approach by the local liver cancer and transplantation conference. The clinical course of all patients with HCC recurrence is listed in S1 Table. Systemic treatment approaches included sorafenib and ramucirumab, or tamoxifen and thalidomide in 13 patients treated before tyrosine kinase inhibitors were approved. Locoregional treatment was performed with transarterial chemoembolization (TACE), percutaneous microwave ablation (MWA) or radiofrequency ablation (RFA), respectively. In patients with resectable tumors, surgery was favored.

## Discussion

Liver transplantation is a curative treatment option in patients with early HCC. However, a considerable number of patients experience HCC recurrence after LT. In the current study, we investigated the incidence of HCC recurrence after LT, in a high MELD area with correspondingly longer waiting times between listing for LT and graft allocation. Furthermore, a thorough risk analysis for HCC recurrence and mortality was performed, with a focus on the relevance of AFP measurements.

A major finding of our analysis is that both overall HCC recurrence rate and patient survival rate were comparable to other reports, including patients transplanted in countries from low MELD areas [9]. While a longer waiting time could be associated with progression and micrometastasis, a prolonged waiting time may also facilitate the detection of biologically more aggressive HCC nodules. As such, patients with rapidly progressive disease are less likely to receive a liver graft, which could explain the aforementioned observation of our study [23].

The second major finding is that AFP measurement is indeed justified in both LT candidates and recipients with HCC prior to LT. In general, serum AFP levels may be associated with HCC tumor mass, but not all HCC patients show high AFP levels, e.g. above 250–400 ng/ml. Besides, mild to moderate AFP elevations may be associated with hepatic necroinflammation independent of malignancy [24]. Thus, it may be assumed that a low AFP serum level prior to LT has limited informative value, but that high AFP levels prior to LT indicate a higher risk for HCC recurrence after LT. Indeed, several studies have reported an increased risk of HCC recurrence after LT in patients with higher AFP levels prior to transplant. Mazzaferro et al. described an HCC recurrence prediction model that includes absolute AFP values prior to LT [25]. Similarly, the MORAL score as published by Brown and colleagues used preoperative AFP values to estimate the HCC recurrence risk after LT [26]. Indeed, we were also able to confirm that AFP levels at LT are significantly correlated with HCC recurrence in our study.

The situation of HCC recurrence after LT, however, differs significantly from HCC development in liver cirrhosis. In patients with advanced liver disease, HCC nodules occur in a necroinflammatory environment of varying severity, mainly within a cirrhotic liver. In contrast, recurrent HCC nodules after LT mainly comprise metastatic lesions to the lungs, liver and bone, and the liver graft is generally not cirrhotic. While we did not observe an association between absolute AFP levels after LT and HCC recurrence, we did find that a rise in serum AFP levels occurred several months before radiological HCC diagnosis and moreover that the ratio of AFP levels at recurrence to AFP levels 3 months before recurrence was predictive for HCC survival after recurrence. Our observation is in concordance with another study, reporting that the steepness (“slope”) of an AFP rise was more important for prediction of recurrence than the value itself [27]. Our finding is clinical meaningful, because it is not only critical to know whether patients are at increased risk for HCC recurrence, but also to detect HCC recurrence as early as possible. Follow-up care of HCC in liver graft recipients has not yet been evaluated in prospective clinical trials. Some centers suggest three-monthly chest CT scans and CT or MRI scans of the liver, and optionally bone scintigraphy, to detect recurrent HCC [19,20,21]. However, performance of repeated CT scans in putatively cured patients are particularly questionable with respect to radiation exposure. The observation that AFP kinetics can be critically informative in the post-LT setting, even when absolute AFP values are comparatively low, may help to identify patients at increased short-term risk of HCC recurrence and thus requiring an intensified surveillance strategy [28–30].

Finally, we recorded treatment strategies and clinical outcomes in patients with recurrent HCC after LT. As no prospective studies are available investigating the optimal treatment of recurrent HCC after LT, treatment strategies are based on approaches in patients with HCC without a history of LT, and retrospective analyses in patients with HCC recurrence after LT [11]. As a consequence, the best treatment strategy for a given patient is currently determined by an individualized and multidisciplinary approach. Treatment options include surgery for localized disease, locoregional treatments such as TACE and radiofrequency ablation (RFA), and systemic treatments such as sorafenib [31].

Ramucirumab is a VEGF receptor-2 antagonist and was investigated in two large placebo-controlled phase III trials as second-line treatment in patients with HCC after treatment with sorafenib [32,33]. We have included two cases of patients with recurrent HCC after LT who were treated with ramucirumab after progression on sorafenib and multimodal treatment. Further trials are needed to evaluate the efficacy and toxicity profile of ramucirumab in this subgroup of patients. Furthermore, experience in the management of patients with recurrent HCC after LT is based on case reports, since these patients were excluded in the clinical trials. The incidence of side effects, toxicities and drug interactions, especially with immunosuppression, is therefore largely unknown.

The major limitation of our study is its limited sample size. Moreover, the retrospective and single-center design has to be taken into account. On the other hand, our cohort covers a period of almost 20 years. Although our data are reliable and the main findings are in concordance with other studies in this field, it is clear that conclusions from our data should be drawn very carefully.

In conclusion, our data indicate that HCC recurrence after LT is not increased, and patient survival is not decreased in a high MELD area with correspondingly longer waiting times for LT. A rise in AFP levels rather than the use of an absolute threshold could help to identify patients at short-term risk for HCC recurrence post LT. Thus, patients with increasing AFP levels after LT should be monitored closely for HCC recurrence and intensified follow-up should be considered.

## Supporting information

S1 Table Clinical course and treatment of patients with recurrent HCC after liver transplantation. (DOCX)

S1 Data Anonymized data set. (XLSX)

## Abbreviations

AFP: alpha-fetoprotein
ALT: alanine transaminase
AST: aspartate transaminase
BCLC: Barcelona Clinic Liver Cancer (group)
BMI: body mass index
CMV: cytomegalovirus
D: donor
ET: Eurotransplant
HBV: hepatitis B virus
HCC: hepatocellular carcinoma
HCV: hepatitis C virus
LT: liver transplantation
MELD: model of end stage liver disease
MMF: mycophenolate mofetil
R: recipient
ULN: upper limit of normal
UNOS: United Network for Organ Sharing
