# Supplementary material for: AFP ratio predicts HCC recurrence after liver transplantation
Source: PLoS One. 2020 Jul 2;15(7):e0235576. doi: 10.1371/journal.pone.0235576 (PMC7332004; doi:10.1371/journal.pone.0235576)
Supplement: S1 Table — (DOCX) [file pone.0235576.s001.docx]

**Supplementary Table 1:** **Clinical course and treatment of patients with recurrent HCC after liver transplantation.**

|  | **1^st^ recurrence** | | | | **2^nd^ recurrence** | | | |
| --- | --- | --- | --- | --- | --- | --- | --- | --- |
|  | **time to recurrence (months)** | **localization** | **treatment** | **clinical course** | **time to recurrence (months)** | **localization** | **treatment** | **clinical course** |
| HCC 183 | 22 | liver | RFA | alive at data base lock |  |  |  |  |
| HCC  106 | 12 | mesentery | surgery and  sorafenib | alive at data base lock |  |  |  |  |
| V21 | 78 | liver | sorafenib | death 1 month after HCC recurrence |  |  |  |  |
| V45 | 8 | liver and lung | sorafenib | after 3 months de novo HCC (liver); after 6 months cerebral metastasis, cerebral bleeding and death |  |  |  |  |
| V33 | 7 | adrenal gland | surgery and radiation | 2 months after surgery multifocal metastatic disease (liver, lung, pleural, bone) |  |  |  |  |
| V40 | 9 | lung | sorafenib | death after 14 months |  |  |  |  |
| V76 | 3 | liver, lung, peritoneum | sorafenib | death after 1 month |  |  |  |  |
| V80 | 15 | liver, lung, peritoneum | tamoxifen, thalidomid and somatostatin | death after 2 months |  |  |  |  |
| V89 | 18 | liver | sorafenib, 5x TACE and 1x TAE |  | 35 | lung, LN (mediastinal) | sorafenib, ramucirumab, radiation | death 9 months after 2^nd^ recurrence |
| V95 | 24 | lung | sorafenib | death after 12 months |  |  |  |  |
| V101 | 22 | abdominal wall | surgery |  | 26 | lung | sorafenib, ramucirumab | progression (bone, lung) |
| V18 | 48 | peritoneum | none | liver failure and death after 3 months |  |  |  |  |
| V6 | 15 | LN | sorafenib | progressive disease after 18 months and lung metastasis | 50 | lung | RFA | death 9 months after RFA |
| V44 | 9 | liver |  | progressive disease and death after 6 months |  |  |  |  |
| V88 | 8 | lung | sorafenib | progressive disease, sorafenib discontinued after 33 months, death 20 months later |  |  |  |  |
| V7 | 31 | liver | TACE, 3x RFA, sorafenib |  | 40 | liver | sorafenib | progressive disease after 14 months with abscesses, 13 months later liver failure and death |
| V29 | 20 | liver, LN | none | death after 14 months |  |  |  |  |
| V52 | 26 | lung, bone | radiation,  sorafenib | death after 9 months |  |  |  |  |
| V4 | 12 | thoracic wall | surgery | 8 months later suspected lung metastasis and death |  |  |  |  |
